# Supplementary material for: CED-3 caspase promotes dismantling but not onset of non-apoptotic linker cell death in C. elegans
Source: bioRxiv. 2025 Oct 15:2025.10.15.682583. Preprint. [Version 1] doi: 10.1101/2025.10.15.682583 (PMC12632748; doi:10.1101/2025.10.15.682583)
Supplement: Supplement 1 [file media-1.pdf]

SUPPLEMENTAL DATA

CED-3 caspase promotes dismantling but not onset of non-apoptotic linker cell death in *C. elegans*

Olya Yarychkivska[1,5], Lena M. Kutscher[1,2,5], Dana Mamriev[3], Betty Ortiz Bido[1], Yun Lu[1], Wolfgang Keil[4], Sarit Larisch[3], Shai Shaham[1,*]

[1]Developmental Genetics, the Rockefeller University, New York, USA

[2]Hopp Children's Cancer Center (KiTZ), Developmental Origins of Pediatric Cancer Group, German Cancer Research Center (DKFZ), D-69120 Heidelberg, Germany.

[3]Cell Death and Cancer Research Laboratory, Department of Human Biology and Medical Sciences, University of Haifa, 31905, Haifa, Israel.

[4]Institut Curie, Université PSL, Sorbonne Université, CNRS UMR168 Laboratoire Physico Chimie Curie, Paris 75005, France

[5]Equal contribution

[*]Correspondence: shaham@rockefeller.edu

SUPPLEMENTAL FIGURE LEGENDS

Sup Fig 1. Nuclear envelope proteins dynamics after cell splitting.

(A) Emerin localization in linker cells during competitive phagocytosis and split stages.

(B) Nucleoporin NPP-1 localization in linker cells during competitive phagocytosis and split stages.

(C) Split linker cells exhibit perinuclear blebbing (arrowhead) and diffuse lamina (arrow). Star marks the nucleus.

Sup Fig 2. Lamina disassembly occurs after phagosome maturation.

(A,B) Confocal live-imaging of a linker cell labeled with *mig-24*p::iBlueberry, lamin endogenously tagged with GFP, and mKate2-PH labeling unsealed phagosome membranes and plasma membrane of the engulfing cells. Asterisk marks the intact lamina of the linker cell nucleus. Arrowhead points to the unsealed phagosome membrane. Arrow points to the engulfing cell membrane.

Sup Fig 3. *ced-3* and *arf-6* genetically interact.

(A-F) Developmental timing of *ced-3(n2452)* animals is similar to wild-type.

(F) Linker cell degradation in indicated genotypes. Strains contain *lag-2p::GFP* linker cell reporter and *him-5(e1490).* Number of animals scored inside bars. Error bars, standard error of the proportion. Fisher's exact test.

Sup Fig 4. *ced-3* expression is lower in the linker cell than in apoptotic cells.

(A) Transgene *ced-3*p::mCherry is expressed in the dying linker cell (triangle) and in the cells dying by apoptosis (arrowheads).

(B) Transgene *ced-3*p::GFP is expressed in the dying linker cell (triangle) and in the cells dying by apoptosis (arrowheads).

Sup Fig 5. Cell cycle genes in LCD.

(A) Linker cell-specific RNAi of cytokinesis (*nmy-2*, *nop-1*) and cell cycle (*plk-1*, *cye-1*) genes.

(B-E') Tubulin/TBA-1 is reorganized during LCD. LCp = *lag-2p*.

SUPPLEMENTAL FIGURES

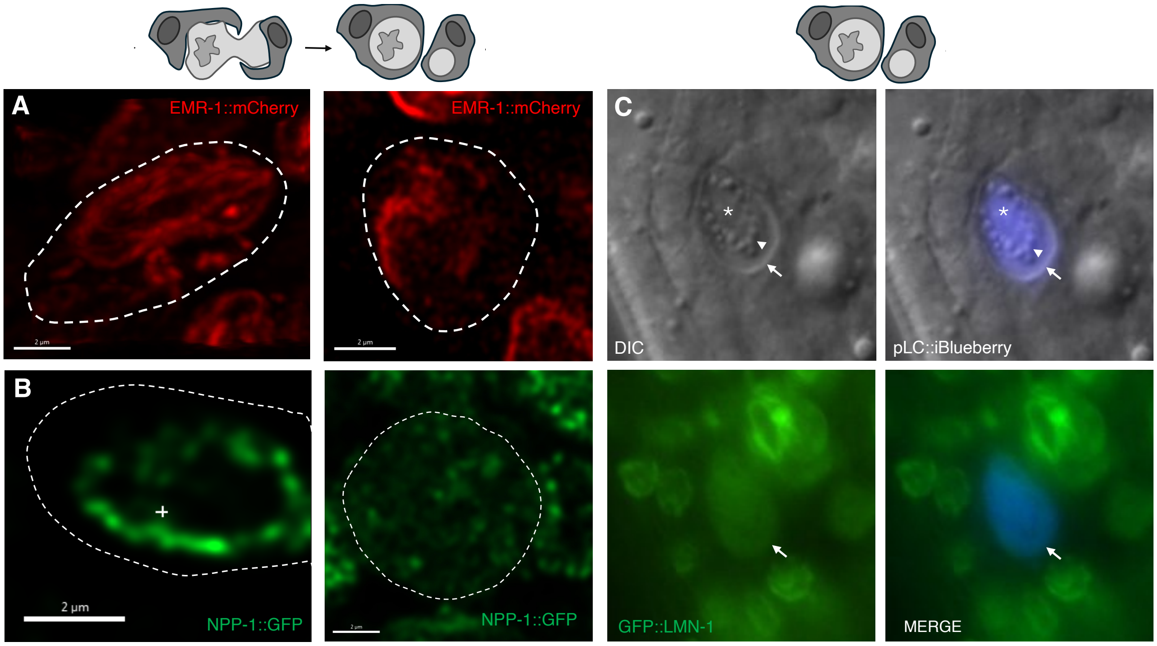

Supplemental Figure 1. Nuclear envelope proteins dynamics after cell splitting.

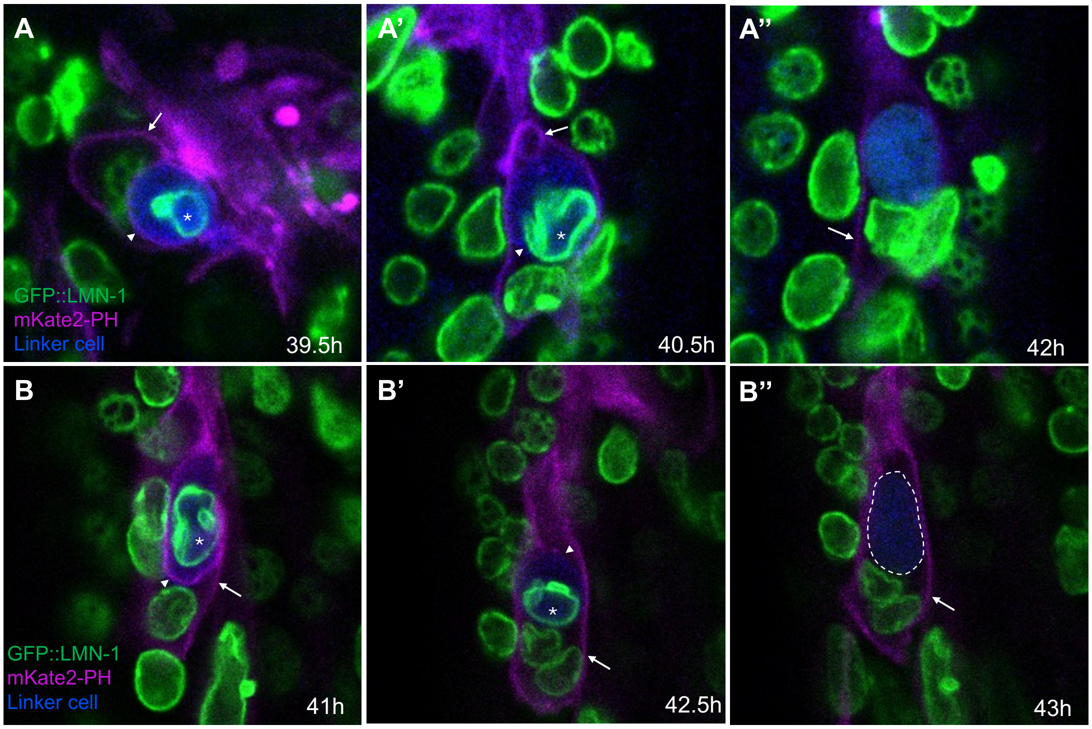

Supplemental Figure 2. Lamina disassembly occurs after phagosome maturation.

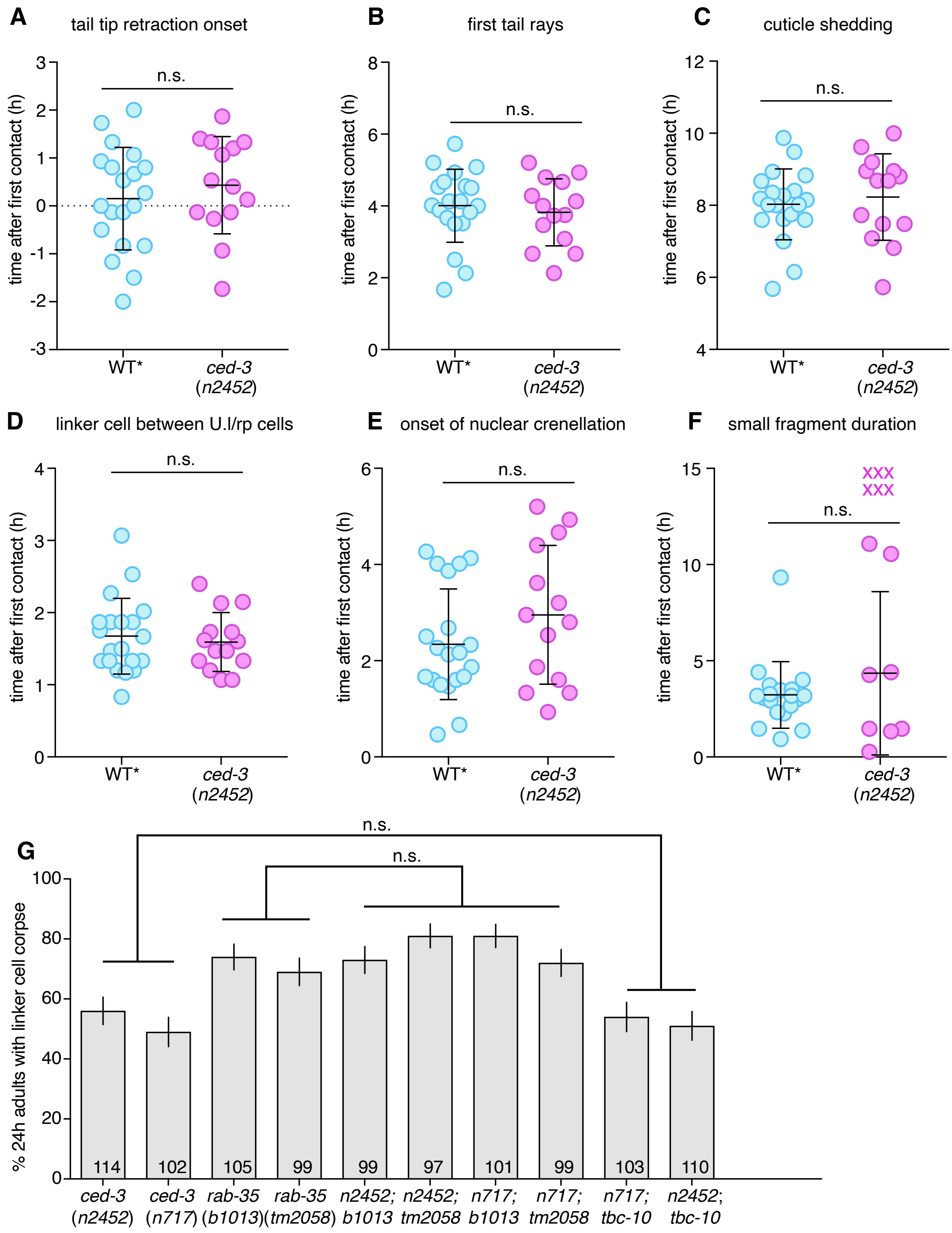

Supplemental Figure 3. Genetic interaction between *ced-3* and *arf-6*.

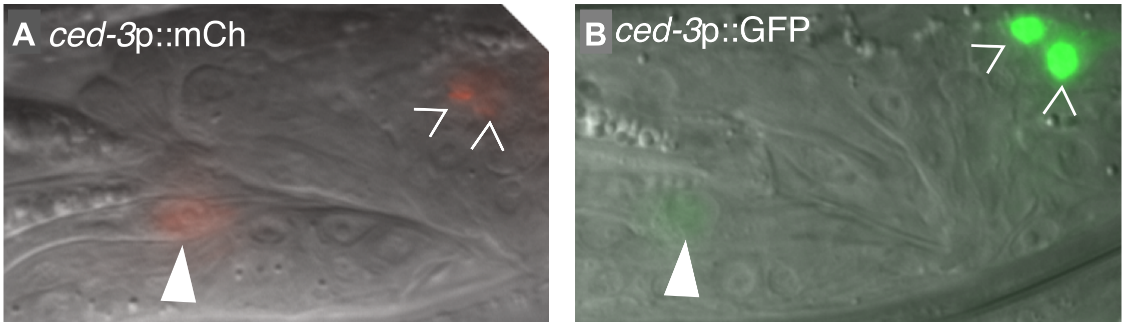

Supplemental Figure 4. *ced-3* expression is lower in the linker cell than in apoptotic cells.

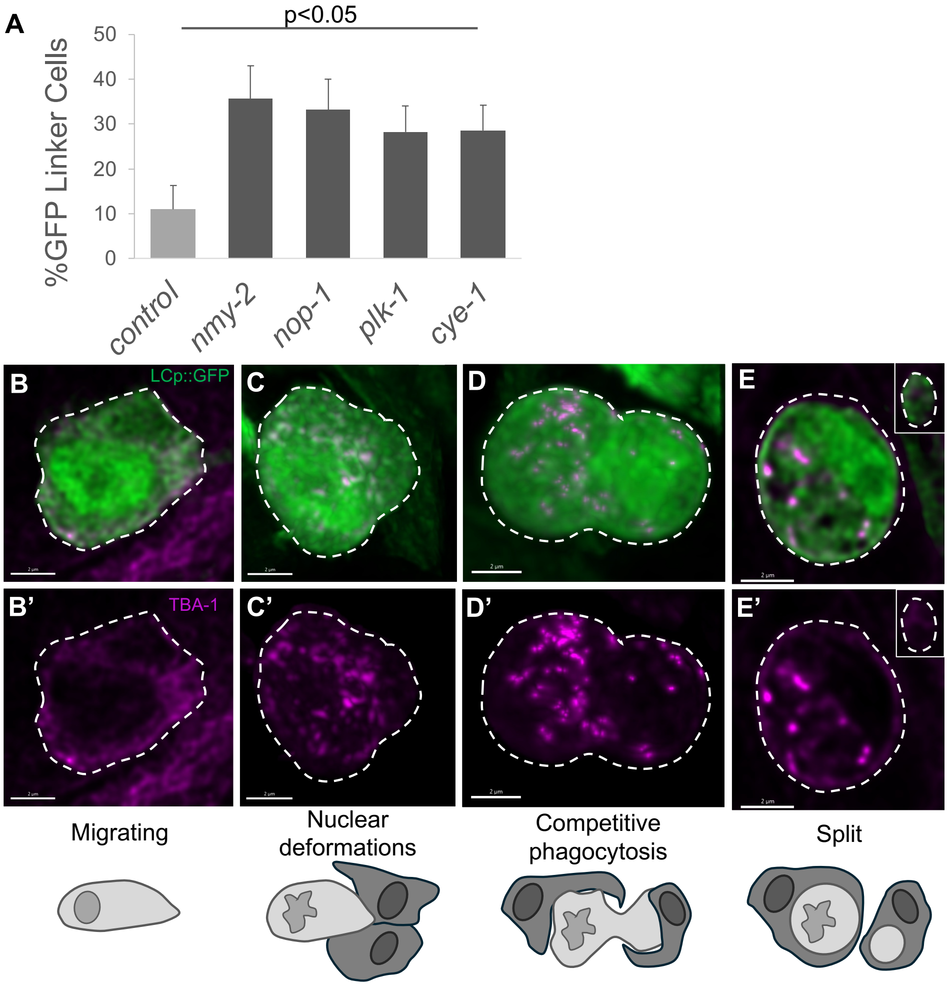

Supplemental Figure 5. Cell cycle genes in LCD.
